# Semiconducting polymer nanoparticles for photothermal ablation of colorectal cancer organoids

**DOI:** 10.1038/s41598-021-81122-w

**Published:** 2021-01-15

**Authors:** Bryce McCarthy, Amit Cudykier, Ravi Singh, Nicole Levi-Polyachenko, Shay Soker

**Affiliations:** 1 Department of Plastic and Reconstructive Surgery Research, Wake Forest School of Medicine, Winston Salem, NC USA; 2 Virginia Tech-Wake Forest School of Biomedical Engineering and Sciences, Wake Forest School of Medicine, Medical Center Boulevard, Winston-Salem, NC USA; 3 Wake Forest Institute for Regenerative Medicine, Wake Forest School of Medicine, Winston Salem, NC USA; 4 Department of Cancer Biology, Wake Forest School of Medicine, Winston‐Salem, NC USA; 5 Comprehensive Cancer Center at Wake Forest Baptist Medical, Medical Center Boulevard, Winston Salem, NC USA

**Keywords:** Cancer, Nanoscience and technology

## Abstract

Colorectal cancer (CRC) treatment is currently hindered by micrometastatic relapse that cannot be removed completely during surgery and is often chemotherapy resistant. Targeted theranostic nanoparticles (NPs) that can produce heat for ablation and enable tumor visualization via their fluorescence offer advantages for detection and treatment of disseminated small nodules. A major hurdle in clinical translation of nanoparticles is their interaction with the 3D tumor microenvironment. To address this problem tumor organoid technology was used to evaluate the ablative potential of CD44-targeted polymer nanoparticles using hyaluronic acid (HA) as the targeting agent and coating it onto hybrid donor acceptor polymer particles (HDAPPs) to form HA-HDAPPs. Additionally, nanoparticles composed from only the photothermal polymer, poly[4,4-bis(2-ethylhexyl)-cyclopenta[2,1-b;3,4-b’]dithiophene-2,6-diyl-*alt*-2,1,3-benzoselenadiazole-4,7-diyl] (PCPDTBSe), were also coated with HA, to form HA-BSe NPs, and evaluated in 3D. Monitoring of nanoparticle transport in 3D organoids revealed uniform diffusion of non-targeted HDAPPs in comparison to attenuated diffusion of HA-HDAPPs due to nanoparticle-matrix interactions. Computational diffusion profiles suggested that HA-HDAPPs transport may not be accounted for by diffusion alone, which is indicative of nanoparticle/cell matrix interactions. Photothermal activation revealed that only HA-BSe NPs were able to significantly reduce tumor cell viability in the organoids. Despite limited transport of the CD44-targeted theranostic nanoparticles, their targeted retention provides increased heat for enhanced photothermal ablation in 3D, which is beneficial for assessing nanoparticle therapies prior to in vivo testing.

## Introduction

Colorectal cancer (CRC) is the fourth most diagnosed cancer in the United States, but the second deadliest owing to limited treatment efficacy when diagnosed at a late stage^1–3^. The standard of care interventions, cytoreductive surgery and chemotherapy, still face clinical limitations; specifically, non-visible micrometastases can evade resection during surgery and are often insufficiently addressed with chemotherapy, leading to patient relapse^4–6^. Together, these complications in treating late stage CRC are reflected in a 5-year survival rate of 14.3%, stressing the importance of novel therapeutic developments to treat CRC^7–9^.

Ablative hyperthermia represents a therapy capable of achieving necrotic cell death through elevated temperature (T > 45 °C), but current methods of ablation cannot safely address diffuse disease^10–12^. A method of targeting ablative hyperthermia to diffuse micrometastases is through the use of photothermal nanoparticles, which are capable of producing heat under photoexcitation^13,14^. Such nanoparticles can be tuned to absorb near-infrared (NIR) light to exploit the maximum penetration of longer wavelengths into tissue within the biological absorption minimum window^15^. Additional functionalization of the nanoparticle surface with a targeting moiety provides a means with which ablative hyperthermia can selectively treat cancer, thus sparing normal tissue^16,17^. Incorporation of a fluorophore creates a theranostic platform that allows for detection and treatment of previously undetectable micrometastases^18^.

**Figure 1:**
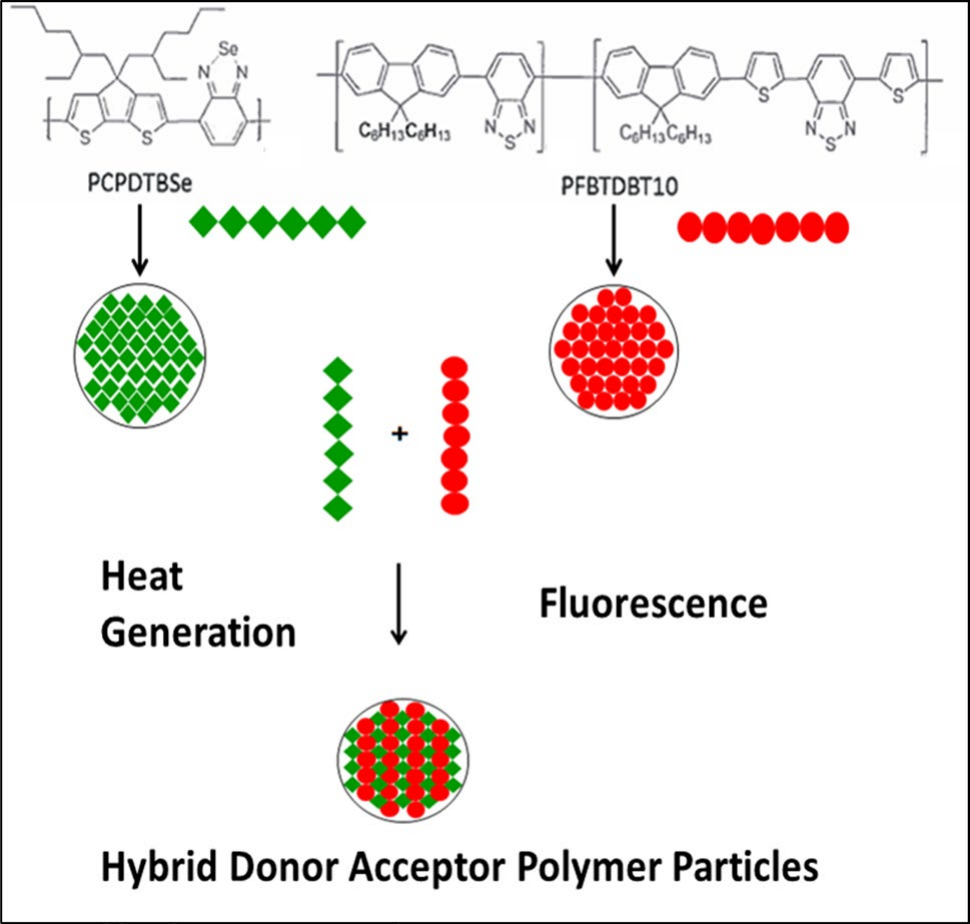
Combination of fluorescent and heat generating polymers to form HDAPPs. The chemical structure of the heat generating polymer, PCPDTBSe (or BSe for short), and the fluorescent polymer, PFBTDBT10 are shown. The polymers are dissolved in an organic solvent and upon addition to water will develop into nanoparticles. The polymers can be used alone to create particles of a single polymer with either heat or fluorescence capacity. Alternatively, ratios of the polymers can be combined to create HDAPPs. Chemical structure prepared using ChemDraw JS, from PerkinElmer (https://www.perkinelmer.com/product/chemdraw-direct-chemdrawdi).

Conjugated donor–acceptor semiconducting polymers are a class of photothermal agents that have recently seen emergence in nanoparticle research for targeted hyperthermia^19^. They have a unique block copolymer structure that makes them amenable for optical tuning by select combination of electron-rich donor and electron-deficient acceptor monomers into the polymer backbone to modify the band gap^20^. In addition to heat production, some conjugated donor–acceptor polymers have been shown to fluoresce as a mode of relaxation^21^. Our group has recently developed a conjugated donor–acceptor polymer nanoparticle composed of the fluorescent polymer poly[(9,9-dihexylfluorene)-*co*-2,1,3-benzothiadiazole] (PFBTDBT10) combined with the photothermal polymer poly[4,4-bis(2-ethylhexyl)-cyclopenta[2,1-b;3,4-b’]dithiophene-2,6-diyl-*alt*-2,1,3-benzoselenadiazole-4,7-diyl] (PCPDTBSe, or BSe for short), as shown in Fig. 1^22^. Termed Hybrid Donor–Acceptor Polymer Particles (HDAPPs), our group has demonstrated the capacity of HDAPPs to reproducibly heat by photoexcitation within the NIR window, generate fluorescence emission with minimal photobleaching within the NIR window (825 nm), remain colloidally stable to indefinite time points, and show minimal cytotoxicity, even up to 1 mg/ml^22–24^. The combination of these characteristics into a single targetable nanoparticle platform demonstrates the utility of HDAPPs as a photothermal theranostic for both detection and ablation of CRC. While ideal for ablative therapy, recent analysis of systemic nanoparticle delivery in vivo demonstrated that the delivery efficiency of nanoparticles to tumors is only 0.7%^25^. Although systemic delivery of chemotherapy is used for disseminated CRC, the blood peritoneal barrier can impede delivery, and thus intraperitoneal perfusion of chemotherapy is often employed. We have shown that HDAPPs can be delivered similarly, using intraperitoneal perfusion that overcomes the biological limitation and improves delivery to the tumor, to serve as a theranostic modality for CRC treatment^24^. However, anisotropic diffusion of nanoparticles within the tumor microenvironment may be a major barrier to therapeutic success, and it is this limitation that the current work has sought to illucidate^26^.

The utility of an ex vivo tumor model to assess and optimize nanoparticle delivery may be fundamental to realizing pre-clinical efficacy with photothermal nanoparticles. Recent advances in biofabrication technologies, such as cell culture systems, and biomaterials have led to the development of three-dimensional (3D) cell culture platforms, such as normal tissue and tumor organoids^27,28^. Culture of cancer cells in 3D extracellular matrix (ECM) confers a more clinically relevant phenotype, with tumor organoids being more physiologically accurate to in vivo pathological and physical characteristics of tumors, compared with traditional two-dimensional (2D) cancer cell culture systems. Additionally, cellular response to 3D culture conditions is known to influence phenotype to match a more clinically relevant profile^29–31^. Furthermore, tumor organoids can be easily controlled in the laboratory to attain the desired tissue characteristics. This method of 3D biofabrication lends to a versatile platform for replicating tumor tissue microstructures in addition to simulating tumor diffusive boundaries^29–31^. These unique properties of tumor organoid cultures illustrate their possible utility as a standard for pre-clinical design and translation of photothermal nanotherapeutics.

In this study, fluorescent HDAPPs and photothermally optimized PCPDTBSe nanoparticles (BSe NPs) were coated with hyaluronic acid (HA) in order to target CD44, which is the HA receptor found to be overexpressed by CRC cells^32^. Targeted fluorescent imaging and photothermal ablation within CRC tumor organoids were then evaluated, demonstrating for the first time the use of a tumor organoid platform to assess targeted semiconducting polymer photothermal nanoparticles within a CRC ECM.

## Results

### Fluorescent HDAPPs and ablative BSe NPs are successfully coated with hyaluronic acid

**Figure 2:**
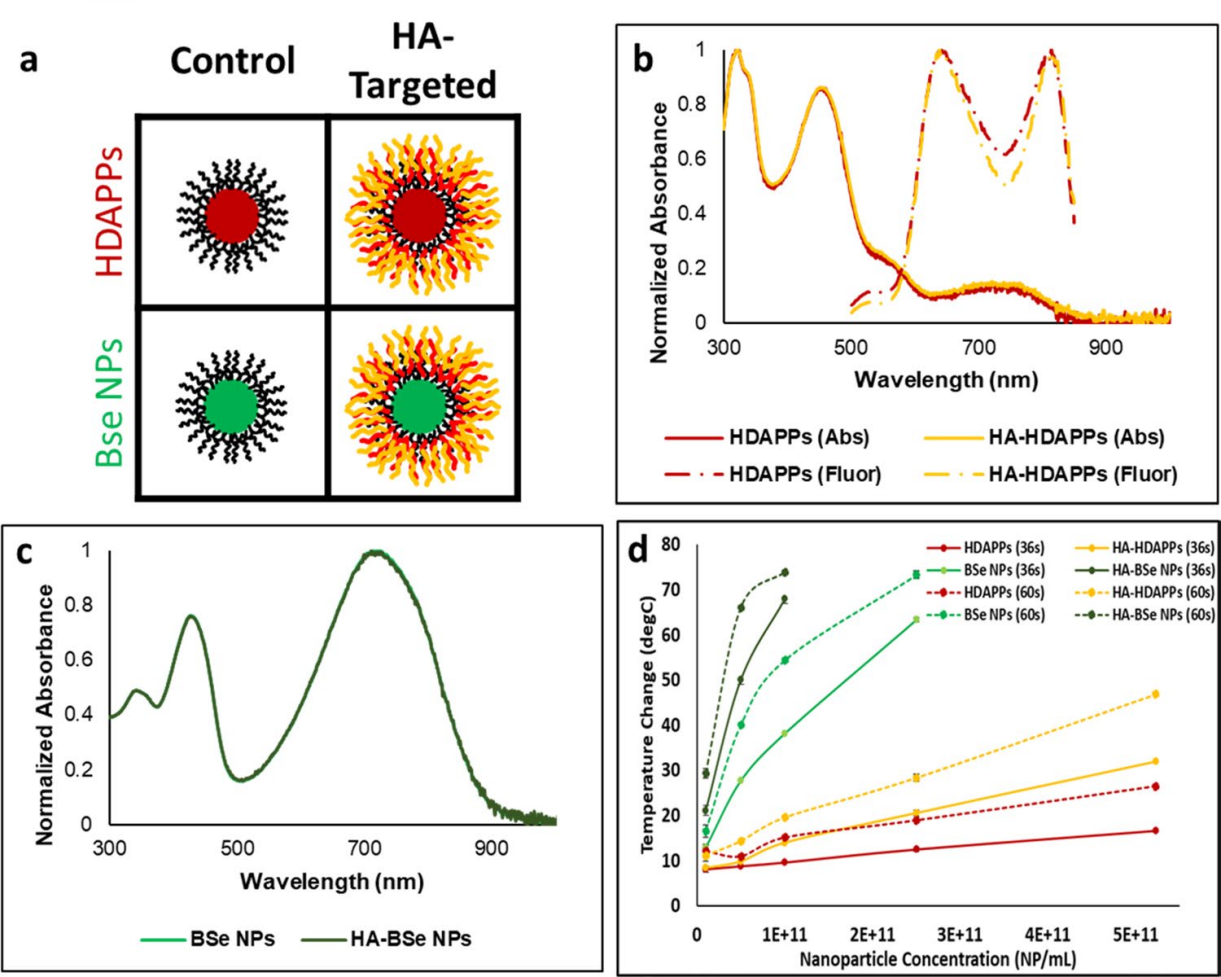
Targeted semiconducting polymer nanoparticle physical characterization. (**a**) Panel of fluorescent HDAPPs and ablative BSe NPs variants used within experimentation, either uncoated, or with HA. (**b**) HDAPPs and HA-HDAPPs UV/Vis absorbance and fluorescence spectra indicate no loss in characteristic activity due to coating with HA. (**c**) BSe NPs and HA-BSe NPs UV/Vis absorbance spectra indicate no loss in characteristic activity due to coating with HA. (**d**) Heating potentials of HDAPPs, BSe NPs, and their HA-coated variants were measured by exposing NPs in a 100 µL volume to 800 nm laser at specified laser durations. This illustrates the capacity of each nanoparticle to reach ablative potential at relatively low concentrations in bulk solution phase. (Error bars reported as s.e.m.).

Fluorescent HDAPPs and ablative BSe NPs were both synthesized and subsequently coated with hyaluronic acid, providing the panel of nanoparticles as presented in Fig. 2a. Neither HDAPPs, nor BSe NPs exhibit significant change in their characteristic absorption or fluorescent spectra following HA coating as shown in Fig. 2b,c. HDAPPs possess a quantum yield of 0.0035 where, following functionalization, HA-HDAPPs possess a quantum yield of 0.0033. As previously reported, HDAPPs both fluoresce (Exc: 452 nm, Em: 645 and 825 nm) and are photothermally activated (Exc: 760 nm) within the near-infrared (NIR) window, between 700 and 900 nm, where tissue absorbs the least^15^. Hydrodynamic diameter as measured by dynamic light scattering (DLS) indicates an initial measurement of 133.6 nm (± 1.3 nm) prior to coating, which then displays a stepwise increase to 149.0 nm (± 1.6 nm) when coated with chitosan and then to 189.1 nm (± 1.3 nm) with the addition of HA (Supplementary Figure S1). Secondary confirmation of diameter by nanoparticle tracking analysis (NTA) indicates average diameters of 85.1(± 2.2 nm) and 134.2 nm (± 7.9 nm) for HDAPPs and HA-HDAPPs, and TEM imaging qualitatively confirms these results (Supplementary Figure S2). Further, NTA size collected in fluorescence mode (133.9 nm ± 5.8 nm) displayed strong agreement with that from the scattering mode. Fluorescence measurement with NTA allows for detection of potentially non-encapsulated fluorophores that would be indicated by a small measured particle size. The size agreement between scattering and fluorescence indicates that the nanoparticles presenting with fluorescence were in fact the isolated HA-HDAPPs and not two separate populations of PFBTDBT10 and PCPDTBSe nanoparticles (Supplementary Figure S2C). This serves as confirmatory evidence that the fluorescent PFBTDBT10 polymer is encapsulated with the photothermal PCPDTBSe in a Pluronic coating, where NTA can then be used to quantify nanoparticle concentration in particles/mL as a function of both scattering and fluorescence.

As an indication of successful surface functionalization, the zeta potential changes from a negative value (− 14 mV ± 0.1 mV), reflecting the base Pluronic F127 coating, to a positive value (41.5 mV ± 0.4 mV), reflecting the chitosan intermediate layer, and finally back to a negative value (− 28.2 mV ± 0.3 mV), suggesting that hyaluronic acid was electrostatically bound to the HDAPPs surface (Supplementary Figure S1). Results of DLS and zeta potential measurements for BSe NPs reveal particles with 131.8 nm (± 0.6 nm) diameter and − 13.8 mV (± 0.3 mV) zeta potential, where coated HA-BSe NPs display a mean diameter of 185.3 nm (± 1.2 nm) and − 13.3 mV (± 0.1 mV) (Supplementary Figure S3). This once again indicates a successful coating process, where BSe NPs are nearly identical by sizing to HDAPPs before and after coating, only containing a different core polymer composition.

Characterization of each nanoparticle’s heating potential reveals that HDAPPs and BSe NPs are capable of generating ablative hyperthermia at concentrations at or above 1 × 10^10^ NP/mL, where BSe NPs generate significantly more heat at each concentration due to their increased molar absorptivity (800 nm) (HDAPPs: 3.08 × 10^11^ mL/(mol*cm); BSe NPs: 3.38 × 10^12^ mL/(mol*cm)) (Fig. 2d). Higher concentrations of BSe NPs and HA- BSe NPs resulted in vaporization of the nanoparticle suspension during laser excitation, and the temperatures could not be accurately measured. Coating with HA for both nanoparticles yields higher temperature changes than those that are non-targeted. Temperature changes of increasing concentration of HDAPPs over time were also evaluated. As shown in Supplementary Figure S4, there is a sharp increase at the initiation of laser stimulation, with a more gradual heating trend during the 60 s exposure. Notably, the 60 s exposure does not result in a thermal equilibrium (plateau of the curve) at any concentration. As a further indication of the retention of the nanoparticles’ heat generating capacity the photothermal conversion efficiency (PTCE) values were found to be 51.2%, 57.1%, 53.17%, and 49.8% for HDAPPs, HA-HDAPPs, BSe NPs and HA-BSe NPs, respectively (Supplementary Figure S5). This close PTCE agreement between all nanoparticle formulations indicates that the BSe polymer PTCE is minimally affected by either coating or core formulation.

### HA-coated NPs display increased binding affinity and ablative potential to CT26 CRC cells

**Figure 3:**
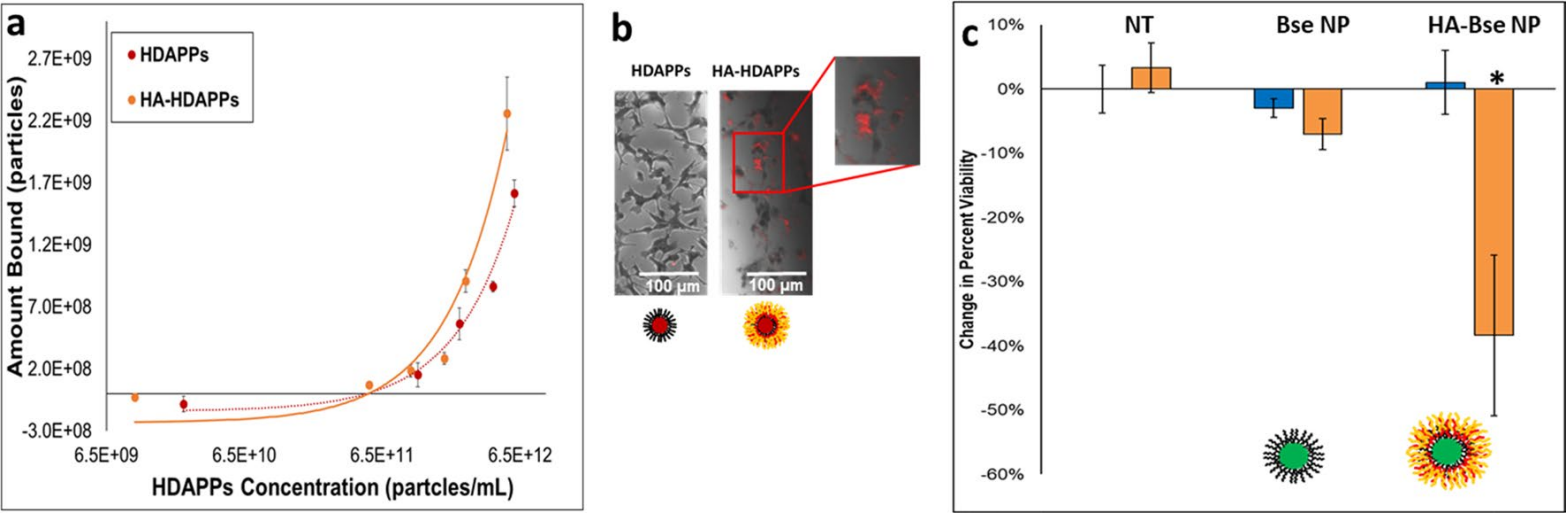
Targeted HDAPPs/BSe NP activity in 2D in vitro culture. (**a**) Fluorescent HA-HDAPPs display an increased binding profile to CT26 cells compared to HDAPPs. (**b**) Qualitative confirmation of HA-HDAPPs binding using by fluorescent imaging registered to the brightfield images of CT26 cells. (**c**) Reduction in the percent of viable cells indicates that only targeted HA-BSe NPs were capable of inducing ablation under laser stimulation. (Error bars reported as s.e.m.).

Binding studies using the fluorescent HA-HDAPPs demonstrated increased binding to CT26 cells in 2D culture relative to non-coated HDAPPs, which could also be visualized by fluorescent and brightfield imaging (Fig. 3b and Supplementary Figure S6). Although we could not reach saturable binding due to the aggregation of high concentrations of the NPs, we observed a significant increase in the binding capacity of HA-HDAPPs compared with HDAPPs (Fig. 3a), as evidenced by the difference in the slope of the curves (Supplementary Figure S7) (p = 0.0012; α = 0.05). Statistical analysis was also done, which confirmed that the difference in the slopes on the binding curve were a result of the HA coating (p = 0.0031; α = 0.05). HA-HDAPPs binding was improved through functionalization with HA, and the maximum amount of HA-HDAPPs was about 2 × 10^9^ NP. This quantity of nanoparticles, in the 0.1 mL volume for in vitro photothermal ablation would yield 1 × 10^10^ NP/mL, resulting in a volumetric temperature change of 11.13 °C with 60 s of laser exposure (from Fig. 2d). Although this temperature change will elevate cells at a baseline temperature of 37 °C into an ablative regime, the temperature attained may not be high enough for effective ablation.

Next, HA-BSe nanoparticles, which should bind similar to HA-HDAPPs, but have superior ablative capacity, were bound to CT26 cells and exposed to 5 W of 800 nm light for photothermal ablation. HA-BSe NPs demonstrated a 38% reduction in cell viability compared with negligible changes in non-coated BSe NPs and nanoparticle-free media exposed to laser. This result suggests that the increased ablation capacity is due to HA targeting that enables efficient cell ablation from rapid localized heating (p < 0.001 for no nanoparticles and p = 0.003 for non-coated BSe NPs; α = 0.05) (Fig. 3c).

### HA-HDAPPs irreversibly sequester at organoid peripheries relative to freely diffusing HDAPPs

**Figure 4:**
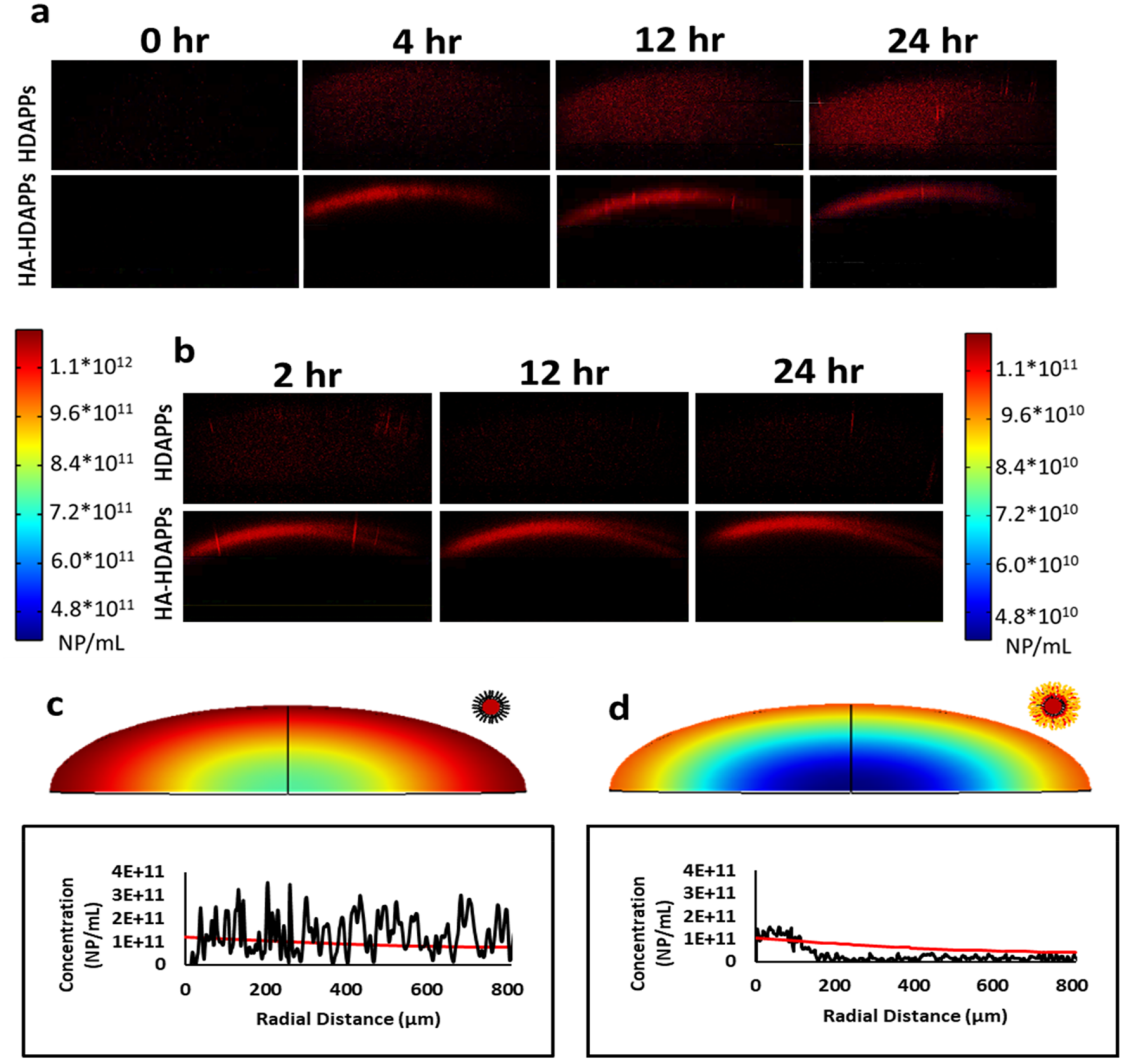
Experimental and computational diffusion profiles. (**a**) Experimental images of fluorescent HDAPPs and HA-HDAPPs diffusing into organoid matrices over a 24 h period and (**b**) experimental images of HDAPPs and HA-HDAPPs diffusing out of organoid matrices over a 24 h period. Computational profiles were calculated for concentration of HDAPPs (**c**) and HA-HDAPPs (**d**), with the red intensity indicating higher concentrations. The red lines indicate the calculated nanoparticle concentration in comparison to the experimentally measured profiles (black lines), measured spatially at 24 h.

Unlike free movement in liquid, the tumor ECM microenvironment limits the efficacy of NP therapeutics by retarding transport processes such as diffusion to become the rate-limiting phenomenon^26^. The 3D tumor organoids allow the direct study of nanoparticle transport within the tumor microenvironment that is not afforded by the 2D culture conditions. Dynamic monitoring of fluorescent HA-HDAPPs and HDAPPs transport within the organoids’ matrix reveals unique diffusive behaviors dependent on nanoparticle coating (Fig. 4a). HDAPPs diffuse throughout the entire organoid depth; however, HA-HDAPPs sequester at the periphery, only penetrating approximately 200 µm of the 915 µm vertical depth of semi-ellipsoidal organoids. Maximum average concentrations reached at 24 h of incubation were 8.57 × 10^10^ (± 3.20 × 10^10^) NP/cm^3^ for HDAPPs and 3.34 × 10^10^ (± 4.60 × 10^9^) NP/cm^3^ for HA-HDAPPs. These values in organoids are approximately two orders-of-magnitude smaller than the concentration in the media surrounding the organoids (5.225 × 10^12^ NP/mL). The concentration of HDAPPs diffusion into organoids after 24 h was determined to be approximately 8.57 × 10^10^ NP/mL (Supplementary Figure S8).

The above results of monitoring diffusion into organoids suggest a potential retention interaction between the ECM microenvironment and HA-HDAPPs. Such a phenomenon is clearly visualized if the nanoparticles are retained within organoids under challenge to diffuse out. Accordingly, we analyzed nanoparticle clearing from the organoids following initial diffusion into organoid’s matrices (Fig. 4b). HA-HDAPPs remain at the organoid periphery and display no drop in concentration within a 24-h clearing period, whereas HDAPPs fully cleared out of organoids within the same 24-h period.

As noted above, the actual concentration within organoids is much lower than the external concentration available. To further establish a predictive dosing model and evaluate nanoparticle transport interactions with organoid matrices, nanoparticle diffusion within a porous matrix was computationally modeled and compared to a representative experimental profile for HDAPPs and HA-HDAPPs. We assumed that the total available concentration of nanoparticles for diffusion into organoids was equal to the edge concentration measured for each representative profile within organoids at 24 h (HDAPPs: 1.18 × 10^11^ and HA-HDAPPs: 1.02 × 10^11^ NP/mL). Comparison of the HDAPPs computational diffusion profile to its experimental profile shows relative agreement, suggesting a diffusive mode of transport (Fig. 4c). In contrast, the computational diffusion profile for HA-HDAPPs displays disagreement with the experimental profile, suggesting that diffusion alone doesn’t explain the HA-HDAPPs’ transport behavior (Fig. 4d). Together, these results indicate that HA-HDAPPs are interacting with the organoid matrix in a manner that restricts their penetration depth.

### HA-coated NPs display increased ablative potential to CT26 CRC organoids

**Figure 5:**
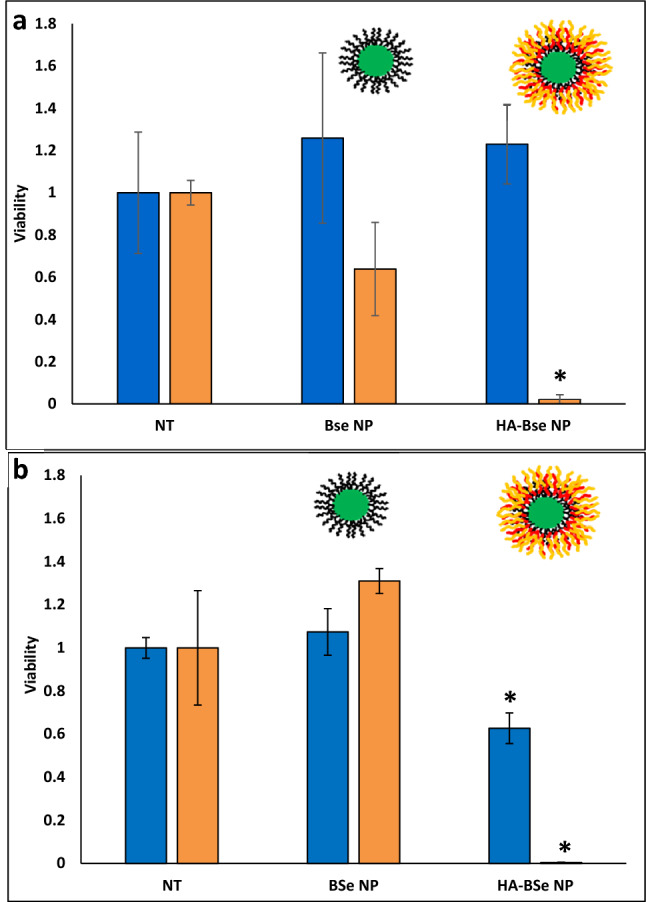
HA-BSe NP photothermal activity in 3D. (**a**) Reduction in organoid viability following treatment with ablative HA-BSe NPs + Laser relative to non-targeted BSe NPs and no nanoparticles (NT-no treatment) with and without laser exposure. *indicates a significant difference between HA-BSe NPs + Laser and both the No Treatment and HA-BSe NPs + No Laser. (Error bars reported as s.e.m.) (**b**) CT26 cells incubated for 1 hr with BSe NPs or HA-BSe NPs prior to the formation of organoids and subsequent laser exposure indicate complete ablation of the cells with HA-BSe NPs plus laser.

Where HA-HDAPPs proved useful in fluorescently monitoring transport, HA-BSe NPs were required to demonstrate photothermal ablation. CT26 CRC organoids were exposed to HA-BSe NPs and uncoated BSe NPs at the same concentration (5.225 × 10^12^ NP/mL) and duration (24 h) as described above for the fluorescent HDAPPs and HA-HDAPPs. The above nanoparticle dose was chosen such that final organoid nanoparticle concentrations would achieve near equivalent temperature changes (BSe NPs: 50.2 °C; HA-BSe NPs: 50.8 °C). Analysis of the ablative capacity of laser-induced HA-BSe NPs revealed almost full elimination of viable cells relative to organoids treated with laser and no nanoparticles (p = 0.006; α = 0.017). In contrast, the non-coated BSe nanoparticles yielded only a 26% reduction in cell viability, which was not statistically significant (p = 0.243; α = 0.05) (Fig. 5a). When BSe NPs or HA BSe NPs were incubated with CT26 cells for one hour prior to their incorporation into an organoid and laser exposure, there was a sufficient concentration of nanoparticles to completely eliminate viable cells as was similar in the 2D binding assay (Fig. 5b). It was also noted that the HA-BSe NPs bound to CT26 cells and in organoids not exposed to laser stimulation also had a reduction on cell viability.

## Discussion

Targeted photothermal nanoparticles present the capacity to localize ablative hyperthermia to diffuse CRC metastases, something unavailable with current hyperthermia interventions utilized clinically^11,12^. When coupled with an imaging agent, detection and treatment becomes simultaneously possible with nanotheranostics. Though a promising treatment modality, photothermal nanoparticles are not approved for clinical use and only one photothermal nanoparticle therapy, AuroLase, is currently undergoing clinical trials of prostate cancer^33–35^. Multiple reports emphasize the role of efficient intratumoral nanoparticle transport and localized, uniform heat delivery as the most important outcomes to achieve complete ablation^26,27,36–38^. From this, we believe 3D tumor constructs to be a fundamental conduit for evaluating nanoparticle utility for ablating micrometastic tumors.

Investigation of photothermal nanoparticles within 3D in vitro tumor constructs is still a field in its infancy^27^. As viable methods of 3D cell culture are established and standardized, the incorporation of these techniques to more rigorously evaluate a nanotherapeutic’s clinical potential represents a valuable tool. The current body of literature surrounding the study of nanotherapeutics in 3D tumor constructs highlights the importance of 3D evaluation of nanoparticle performance^27^. This is most apparent in a study of paclitaxel-conjugated micelles for prostate cancer where 2D assay indicated no benefit of micelles over free drug, but micelles instead proved to be more efficacious than free drug in 3D spheroids^39^. As is most evident in the work presented here, our current dependency on standard tissue culture methods in plastic dishes cannot sufficiently model nanoparticle transport behavior in a perfusion model of therapeutic delivery.

We demonstrate here a next-generation photothermal nanoparticle platform combined with the well-established HA targeting mechanism^40–42^. Optical characterization of both HDAPPs and HA-HDAPPs shows their characteristic photothermal excitation and fluorescent emission within the biologically optimal NIR window^22^. In addition to the benefit of NIR-availability, both HDAPPs and HA-HDAPPs are non-cytotoxic, colloidally stable in aqueous solutions, and reproducibly heat and fluoresce, serving as an ideal theranostic for tumor imaging and ablation^22,24^. HA-HDAPPs demonstrated increased binding potential relative to non-targeted HDAPPs with a 1 h binding incubation; however, there was insufficient binding of HA-HDAPPs (a maximum of 2 × 10^9^ NP) to achieve ablation, leading to the negative results in Supplementary Figure S9. This result is mainly due to the fact that when the HDAPPs NPs are utilized, an insufficient amount of the photothermally active agent is delivered to the CT26 CRC cells (the BSe polymer, which comprises only 1/10 of the HDAPPs formulation). Our team has previously evaluated the optimal ratios of the fluorescent polymer PFBTDBT10 and PCPDTBSe in a nanoparticle formulation to maximize the fluorescent component, while still offering heating for mild hyperthermia^22^. Unfortunately, combining these two polymers leads to chain interaction and subsequent fluorescence quenching. To address this deficiency, we developed HA-BSe NPs based on our previously formulated BSe NP^43^. Successful reduction in 2D viability (Fig. 3c), using HA-BSe NPs suggested that sufficient binding was in fact achieved for efficacious photothermal therapy. Future directions of the HDAPPs formulation will include molecular spacers to minimize PFBTDBT10 and PCPDTBSe polymer chain interactions, thus reducing fluorescence quenching and allowing for increased amounts of the heat generating polymer to be incorporated in HDAPPs.

This work represents the first exploration into the photothermal activity of organic semiconducting polymer nanoparticles in bioengineered 3D tumor tissue constructs (organoids). As is reinforced by other reports on photothermal nanoparticle activity in 2D versus 3D, our nanoparticles failed to achieve ablation in 3D organoids incubated for only 1 h, although 1 h was sufficient for successful ablation in 2D (Supplementary Figure S10 and Fig. 3c)^27^. These results indicate that the rate-limiting process of our therapy is intratumoral diffusion. Confocal monitoring of fluorescent HDAPPs and HA-HDAPPs within organoid matrices void of cells supports this, where HDAPPs were undetectable at 1 h of diffusion and only start to become visible at 4 h (Fig. 4 and Supplementary Figure S8). Uncoated HDAPPs display uniform diffusion profiles throughout the organoids, contrary to multiple reports that indicate non-functionalized nanoparticles are limited to the tumor periphery^27,44,45^. As another contradiction to previously published results, our functionalized HA-HDAPPs do not diffuse beyond the organoid periphery, where others reported greater penetration with functionalization^27,44,45^. When compared to the computationally determined diffusion profile, HA-HDAPPs are experimentally underrepresented within the organoid core, suggesting a matrix interaction that sequesters and retains the nanoparticles at the periphery.

The tumor organoid platform is presented here as a means to test photothermal treatment parameters in order to realize therapeutic efficacy that is both clinically and physiologically relevant. Tumor organoid models recapitulate the physical boundaries that cannot be replicated in 2D culture (e.g., diffusion hindrance), where we demonstrate methods of quantifying some therapeutic barriers due to the presence of an ECM^27^. We showed that HA-HDAPPs display enhanced binding to CT26 CRC cells in 2D culture, yet they have poor penetration into tumor organoids. According to prior reports that indicate peripheral immobilization to micrometastatic tumor models may be all that is necessary to deliver sufficient thermal dose from photothermal nanoparticles, the 3D tumor organoid model confirms this^27,46^. We demonstrate here that the most important parameters for efficacious treatment are photothermal agent availability within the nanoparticle formulation, diffusion time, nanoparticle surface functionalization, and laser duration.

As organoid models become more complex, we envision the utilization of tumor organoids to quantify additional therapeutic-limiting effects beyond what is demonstrated here (e.g., the addition of stromal co-culture or tumor interstitial pressure gradients)^47,48^. For CRC, the clinical relevance of evaluating HDAPPs in tumor organoids is most prominent in the similarity between tumor organoids and micrometastases that are missed by cytoreduction^4^. With a clinically relevant model such as the tumor organoid, the capacity for high throughput analysis is important in the context of pre-clinical candidacy screening of theranostic nanoparticles.

## Conclusions

This study represents the first investigation of semiconducting polymer nanoparticles in a 3D organoid model of photothermal ablation. We demonstrated that transport character in 3D has a strong influence on nanoparticle delivery. Following optimization we demonstrated successful targeted ablation in 3D, which required both increased dosing and delivery time. These results emphasize the observation that therapeutic design and dosing in standard 2D culture conditions often does not translate clinically, where we suggest the 3D tumor organoid to fill this experimental gap for novel photothermal nanoparticles such as the HDAPPs platform used here.

## Materials and methods

### Materials

4H-cyclopenta[1,2-b:5,4-b’]dithiophene, 4,7-Dibromo-2,1,3-benzoselenadiazole, 4,7-bis(5-bromo-2-thienyl)-2,1,3-benzothiadiazole, and 2,7-Bis(4,4,5,5-tetramethyl-1,3,2-dioxaborolan-2-yl)-9,9-dihexylfluorane were purchased from TCI America. Pluronic F127, tetraethylammonium hydroxide, 4,7-dibromo-2,1,3-benzothiadiazole, 4,7-Bis(2-bromo-5-thienyl)-2,1,3-benzothiadiazole, tetrakis(triphenylphosphine)palladium(0), Tris base, oxaliplatin, ethylenedinitrilotetraacetic acid (EDTA), Triton X-100, deoxycholic acid, and chitosan (medium molecular weight, 190–310 kDa) were purchased from Sigma Aldrich. Sodium Hyaluronate (60 kDa) for nanoparticle functionalization was purchased from Lifecore Biomedical. Heprasil Hyaluronan Acid for organoid matrix was purchased from ESI-BIO. 2-Hydroxy-4′-(2-hydroxyethoxy)-2-methylpropiophenone photoinitiator was purchased from Sigma Aldrich (410896). Methacrylated Type I Collagen for organoid matrix was purchased from Advanced Biomatrix.

1X Dulbecco’s Modified Eagle Medium (DMEM) supplemented with 4.5 g/L D-glucose, L-glutamine (1X), 110 mg/mL sodium pyruvate, penicillin and streptomycin (1X), 10% fetal bovine serum, and with or without 400 µg/mL G418 (neomycin) and Fluorobrite DMEM supplemented with 4.5 g/L D-glucose, L-glutamine (1X), 110 mg/mL sodium pyruvate, penicillin and streptomycin (1X), and 10% fetal bovine serum were purchased from Gibco.

### Cell culture

CT26.WT-Fluc-Neo cells, a mouse colorectal carcinoma cell line transduced with firefly luciferase flanked by a neomycin resistance gene, were purchased from Imanis Life Sciences (CL043), and cultured in DMEM.

### Formation of 3D tumor organoids

CT26 tumor organoids were formed by suspending 100,000 CT26 cells in 10 μL of a heprasil hyaluronan acid/methacrylated collagen solution (0.5 mg/mL heprasil/4.1 mg /mL collagen) in a 96-well plate, and then exposing them to UV light (BlueWave 75, Dymax) for 2 s to crosslink them^49^. Heprasil (2 mg/mL) was prepared by re-suspending in 1 mL sterile MilliQ H_2_O containing 0.1% of the photoinitiator. Collagen was prepared according to the manufacturer’s protocol. Once seeded, tumor organoids were incubated in Fluorobrite DMEM.

### Formation of CD44-targeted HDAPPs and BSe nanoparticles

PFBTDBT10 and PCPDTBSe polymers were synthesized as described previously^22^.

HDAPPs were prepared by nanoprecipitation according to a modified protocol from our group^22^. PFBTDBT10 (0.93 mg/mL) and PCPDTBSe (0.1 mg/mL) in 1 mL of THF was rapidly injected into an 8 mL solution of 1 mg/mL Pluronic F127 under horn sonication (1 min, 20% amplitude). Following nanoprecipitation, THF was evaporated off by stirring and heating at 55 °C for 1.5 h. To sterilize, HDAPPs were then autoclaved and isolated by centrifugation.

To target the CD44 receptor, HDAPPs were coated with 60 kDa hyaluronic acid through layer-by-layer electrostatic deposition by modifying our published protocol in accordance with other work, using chitosan as an intermediate layer between the HDAPPs and HA^24,40,50^. First, 350 μL HDAPPs were added to a 5 mL solution of chitosan (3 mg/mL) in 2% acetic acid (filtered with 0.22 μm filter) and stirred for 1 h, followed by centrifugation to remove nanoparticle aggregates. Washed chitosan-coated-HDAPPs were then added to a 10 mL solution of 60 kDa sodium hyaluronate (100 μg/mL) in Milli-Q water, stirred for 1 h, and centrifuged to yield isolated HA-HDAPPs.

PCPDTBSe only nanoparticles were formed by instead using 2 mg/mL PCPDTBS in THF during nanoprecipitation, and coated with HA, as described above, to yield HA-BSe NPs. Following synthesis, HDAPPs, HA-HDAPPs, BSe and HA-BSe NPs were characterized with ultraviolet/visible (UV/Vis) spectroscopy for absorbance (UV5Nano, Mettler Toledo) and fluorescence emission spectra (Infinite M200 Microplate Reader, TECAN), dynamic light scattering (DLS) and zeta potential (Zetasizer Nano ZS, Malvern Panalytical), nanoparticle tracking analysis (NTA) using a 405 nm laser in both scattering and fluorescence mode (NanoSight NS500, Malvern), and transmission electron microscopy (TEM) (FEI Tecnai BioTwin Transmission Electron Microscope).

### Nanoparticle heat generation

Heat generated by HDAPPs, HA-HDAPPs, BSe NPs, or HA-BSe NPs was determined by exposing 100 μL nanoparticle dilutions in water (5.225 × 10^11^, 2.5 × 10^11^, 1 × 10^11^, 5 × 10^10^, and 1 × 10^10^ NP/mL) to 800 nm continuous wave laser at 5 W for 36 s (used for 2D culture) or 60 s (used for organoids) with a 1 cm^2^ spot size (K-Cube, Summus Medical Laser), according to our previous publications^22,24,51^. Solution temperatures before and after exposure were recorded using a Neoptix Nomad fiber optic thermometer. To evaluate the temperature change over time (60 s interval), 200 µl volumes of HDAPPs at different concentrations were evaluated during laser exposure (3 W, 800 nm) using a Neoptix Nomad fiber optic thermometer placed in the solutions.

### HA-HDAPPs binding to CT26 colorectal cancer cells in 2D culture

HDAPPs and HA-HDAPPs binding affinity to CT26 CRC cells was determined by seeding cells at a density of 18,750 cells/cm^2^ in a 96-well plate. Following incubation for 24 h, cells were treated with 100 μL of HA-HDAPPs or 100 μL of HDAPPs for 1 h at 4 °C at the following concentrations in DMEM: 1.18 × 10^13^, 5.9 × 10^12^, 4.1 × 10^12^, 2.4 × 10^12^, 1.2 × 10^12^, and 0 NP/mL for HDAPPs and 5.2 × 10^12^, 2.6 × 10^12^, 1.8 × 10^12^, 1.0 × 10^12^, 5.2 × 10^11^, and 0 NP/mL for HA-HDAPPs, after which they were washed with HEPES-buffered saline, lysed with Tris-Triton buffer, and the fluorescence intensity was measured with excitation at 450 nm and emission read at 650 nm (Infinite M200 Microplate Reader, TECAN).

Nanoparticle binding was quantified using a calibration curve prepared by measuring fluorescence intensity at 650 nm of 100 μL standard solutions of known HDAPPs and HA-HDAPPs concentrations in Tris-Triton buffer after excitation at 450 nm. The following concentrations were used: 7.1 × 10^10^, 2.4 × 10^10^, 2.4 × 10^9^, 2.4 × 10^8^, 2.4 × 10^7^, 2.4 × 10^6^, and 0 NP/mL for HDAPPs and 3.1 × 10^10^, 1.1 × 10^10^, 1.1 × 10^9^, 1.1 × 10^8^, 1.1 × 10^7^, 1.1 × 10^6^, and 0 NP/mL for HA-HDAPPs.

To qualitatively display HA-HDAPPs binding, 1.65 × 10^12^ NP/mL of HDAPPs or 7.32 × 10^11^ NP/mL HA-HDAPPs were incubated with cells for 60 min at 4 °C, washed twice with cold HEPES-buffered saline, fixed in formaldehyde, stained with methylene blue, and then imaged via fluorescence microscopy with a Texas red channel. To confirm that nanoparticles were not adhering to the plastic well, similar procedures were followed in wells without cells.

### Targeted HA-BSe NP ablation of CT26 colorectal cancer cells in 2D culture

CT26.WT-Fluc-Neo cells were seeded at a density of 18,750 cells/cm^2^ in a 96-well and treated with 100 μL of 5.225 × 10^12^ particles/mL or no treatment control (Fluorobrite DMEM) for 1 h at 4 °C to achieve binding. Following treatment, cells were washed with HEPES-buffered saline + 0.1% bovine serum albumin (BSA) to remove non-specifically bound nanoparticles and exposed to 5 W, 800 nm laser for 36 s with a 1 cm^2^ spot size (cell temperature was regulated at 37 °C during laser exposure by placing the plate in a PCR heating block maintained at 37 °C). Twenty-four hours following treatment, cells were stained with Trypan Blue and assayed for viability using the Countess automated cell counting system.

### HDAPPs diffusion into 3D tumor organoid extracellular matrices

Organoid extracellular matrices without cells were formed by crosslinking 10 μL of a heprasil hyaluronan acid/methacrylated collagen solution in a 96-well plate such that the geometry formed a semi-ellipsoid with its planar surface in direct contact with the plastic well and its curved surfaced exposed. They were then incubated for 0, 4, 12, and 24 h with HDAPPs or HA-HDAPPs (5.225 × 10^12^ particles/mL) in Fluorobrite DMEM. At each time point organoids were washed with HEPES-buffered saline (250 mM) + 0.1% BSA and imaged using a Leica TCS LSI Macro Confocal 490-1 Microscope (using an excitation of 488 nm and emission filter from 600 to 800 nm). Nanoparticle concentration as a function of z-penetration depth was determined by selecting three representative lines drawn from the organoid boundary to the center and plotting average fluorescence intensity as a function of depth for each time point. Nanoparticle concentration as a function of depth was then correlated to fluorescence intensity using standards (described below). Average nanoparticle concentration within the whole organoid was also determined by taking the average concentration measured along the lines for each time point. Following 24 h of diffusion into organoids, HDAPPs and HA-HDAPPs diffusion out of organoids into nanoparticle-free Fluorobrite DMEM was measured at 2, 12, and 24 h using the same protocol.

Fluorescence standards for HDAPPs and HA-HDAPPs concentrations within tumor organoids were made by suspending respective nanoparticles within tumor organoids at the following concentrations and imaging as previously described: 1.178 × 10^12^, 1.178 × 10^11^, 1.178 × 10^10^, 1.178 × 10^9^, and 0 NP/mL for HDAPPs and 5.225 × 10^11^, 5.225 × 10^10^, 5.225 × 10^9^, 5.225 × 10^8^, and 0 NP/mL for HA-HDAPPs.

### Computational modeling of microscale diffusive transport of nanoparticles into organoid matrices

Theoretical modeling of HDAPPs diffusion into 10 µL organoid geometries at 24 h was performed utilizing COMSOL according to a previously validated model^52^. Organoid geometry for input into the model was experimentally determined by caliper measurement of three organoids. Nanoparticle diffusivity was estimated using the Stokes–Einstein equation (Eq. 1) for HDAPPs and HA-HDAPPs (133.6 and 189.1 nm diameter, respectively) and corrected for diffusion of rigid spheres into a porous matrix using the Ogston model (Eq. 2). A weighted average of collagen and HA fiber volumetric fractions (*φ* = fiber concentration*fiber specific volume) and radii was used to determine the Ogston model correction factor, then the mass transport equation (Eq. 3) was solved as a time-dependent process excluding convective transport and isolating diffusion to the radial direction. Parameters used for modeling are outline in Supplementary Table S1 provided in the supplemental information. With the given assumptions, the following equations of mass transport were utilized within finite element analysis software (COMSOL)^52,53^.

1 $$ D_{0} = \frac{{k_{B} T}}{{6\pi \eta r_{p} }} $$

where Eq. (1) is the Stokes–Einstein relationship for the diffusivity (*D*_*0*_) of a spherical body as a function of *k*_*B*_ (Boltzman constant), *T* (temperature), *η* (solvent viscosity), and *r*_*p*_ (particle radius).

2 $$ D_{int} = D_{0} e\left[ {\left( { - \sqrt {\varphi_{interstitial} } } \right)*\frac{{r_{p} }}{{r_{f} }}} \right] $$

where Eq. (2) (Ogston model) is a correction for diffusivity in a porous matrix (*D*_*int*_) with *φ*_*interstitial*_ (volume fraction of interstitial matrix) and *r*_*f*_ (fiber radius).

3 $$ \frac{{\partial C_{interstitial} }}{\partial t} = D_{int} \left( {\frac{1}{{r^{2} }}\frac{\partial }{\partial r}\left( {r^{2} \frac{{\partial C_{interstitial} }}{\partial r}} \right)} \right) $$

where Eq. (3) represents the governing mass transport equation measuring time-dependent *C*_*interstitial*_ (interstitial concentration) as a function of *D*_*int*_ (diffusivity in a porous matrix) and *r* (radius).

### Targeted HA-BSe NP ablation of CT26 colorectal cancer cells in tumor organoids

#### Diffusion dependent

Tumor organoids were incubated for 24 h and then exposed to no nanoparticles, BSe NPs or HA-BSe NPs (5.225 × 10^12^ particles/mL) in DMEM for 24 h. Organoids were then washed and treated with an 800 nm continuous wave laser at 5 W for 60 s with a 1 cm^2^ spot size (K-Cube, Summus Medical Laser) in 100 µL of nanoparticle-free DMEM without phenol red. Twenty-four hours following laser ablation, organoid viability was assessed using CellTiter-Glo 3D Cell Viability Assay, where luminescence was measured in a Varioskan Lux plate reader (Thermo Fisher).

#### Diffusion independent

In order to isolate and quantify the resistance that the 3D ECM confers on CT26 cells to photothermal therapy via HA-BSe NPs in comparison to 2D culture without diffusive hindrance, the following protocol was performed. CT26 cells were harvested from non-tissue culture treated culture plates by EDTA lifting to preserve CD44 receptor integrity. Following lifting, cells were incubated in suspension with 5.225 × 10^12^ particles/mL BSe NPs, HA-BSe NPs, or no treatment for 1 h at 4 °C in. Cells were then isolated by centrifugation and washed to remove non-bound particles. Isolated cells with bound nanoparticles were then used to form tumor organoids and after 24 h of incubation organoids were exposed to an 800 nm continuous wave laser at 5 W for 250 s with a 1 cm^2^ spot size (K-Cube, K-Laser), and 24 h later organoid viability was assessed.

### Statistical analysis

All experiments were performed in triplicate, excluding the diffusion assays which were performed in duplicate for diffusion in and singlicate for diffusion out. Statistical analyses were performed using two-way ANOVA for all ablation assays with post hoc analyses (Fisher’s LSD for 2D and Holm-Sidak for 3D) and ANCOVA for binding profiles.

## Supplementary Information

Supplementary Information.

## Data Availability

All data generated or analyzed during this study are included in this published article (and its Supplementary Information files).
