# Supplementary material for: Semiconducting polymer nanoparticles for photothermal ablation of colorectal cancer organoids
Source: Sci Rep. 2021 Jan 15;11:1532. doi: 10.1038/s41598-021-81122-w (PMC7810691; doi:10.1038/s41598-021-81122-w)
Supplement: Supplementary file 1 — Supplementary Information. [file 41598_2021_81122_MOESM1_ESM.docx]

**Semiconducting Polymer Nanoparticles for Photothermal Ablation of Colorectal Cancer Organoids**

Bryce McCarthy^1,2,3^, Amit Cudykier^3^, Ravi Singh^4,5^, Nicole Levi-Polyachenko*^1,2,5^, Shay Soker*^2,3,4,5^

**Affiliations:**

1) Department of Plastic and Reconstructive Surgery Research, Wake Forest School of Medicine, Winston Salem, NC, USA

2) Virginia Tech-Wake Forest School of Biomedical Engineering and Sciences, Wake Forest School of Medicine, Medical Center Boulevard, Winston-Salem, NC, USA

3) Wake Forest Institute for Regenerative Medicine, Wake Forest School of Medicine, Winston Salem, NC, USA

4) Department of Cancer Biology, Wake Forest School of Medicine, Winston‐Salem, NC, USA

5) Comprehensive Cancer Center at Wake Forest Baptist Medical, Medical Center Boulevard, Winston-Salem, NC, USA

**Corresponding authors*:** [ssoker@wakehealth.edu](mailto:ssoker@wakehealth.edu)

nlevi@wakehealth.edu

**Supplementary Methods**

*Nanoparticle Fluorescence Quantum Yield*

Fluorescence quantum yield was determined relative to Rhodamine 6G and Fluorescein dyes according to a previously established protocol.^1^ The fluorescence quantum yield of each reference was determined relative to the other using the following formula: Φ = Φ_R_(m/m_R­­­_)(η^2^/ η_R_^2^), where Φ is the fluorescence quantum yield, m is the slope of the plot of integrated fluorescence intensity versus absorbance, and η is the solvent refractive index (ethanol and 0.1 M NaOH: η = 1.36). The subscript “R” represents a reference parameter. Following calibration, HDAPPs and HA-HDAPPs fluorescence quantum yield was determined by measuring their respective fluorescent spectra (exc. 452 nm; em. 600-840 nm) in water at multiple dilutions below 0.1 peak absorbance (Varioskan Lux plate reader, Thermo Fisher). The fluorescence quantum yield was determined as outlined above relative to each reference.

*Nanoparticle Photothermal Conversion Efficiency*

Photothermal conversion efficiencies of HDAPPs, HA-HDAPPs, BSe NPs, and HA-BSe NPs were determined by irradiating 3.9 mL of 1.18*10^11^ NP/ mL (HDAPPs), 5.23*10^10^ NP/ mL (HA-HDAPPs), 7.47*10^9^ NP/ mL (BSe NPs), and 3.31*10^9^ NP/ mL (HA-BSe NPs) solutions in a 10 mm pathlength quartz cuvette (QS High Precision Cell, 100-10-40). Nanoparticle solutions under continuous stirring were exposed to 800 nm laser (1 cm^2^, 3 W) for 60 minutes while temperature was monitored at a 1 second sampling rate with a Neoptix NOMAD-Touch Optical Thermometer. Following irradiation, the solution temperatures were monitored until the initial, ambient temperature (T_amb_) prior to irradiation was achieved. To calculate the photothermal conversion efficiencies, first, the characteristic time constant for cooling (τ_s_) was determined by plotting the non-dimensionalized temperature driving force (**Eq. S1**) vs. time and taking the negative reciprocal of the slope of a linear fit of the data.

$\theta= \frac{T_{amb}-T}{T_{amb}-T_{max}}$ (**Eq. S1**)

To determine the heat loss due to external heat flux, the linearized form of Newton’s Law of Cooling is employed as in **Equation S2**.

$Q_{ext}=hA(T-T_{amb})$ (**Eq. S2**)

To solve for the heat-transfer coefficient across the entire area of flux (hA), **Equation S3** is utilized.

$\tau_{s}=\frac{\sum_{i} m_{i}C_{p,i}}{hA}$ (**Eq. S3**)

Under the assumption that the nanoparticles are a negligible component of mass (m_np_ < 0.005 % total mass), the system properties are defined for water with a heat capacity (C_p,H2O_) of 4.18 kJ/kg*K and a total mass of 3.9 g. The hA term is then utilized to solve the photothermal conversion efficiency at steady state laser irradiation, where the heat dissipation due to water absorption (Q_0_) was determined to be 0.0692 W and subtracted from the overall heat generation according to **Equation S4**. Q_I­_ is the energy influx due to nanoparticle absorption and is described by **Equation S5**, where I is the laser irradiation power, A_λ_ is the absorption of nanoparticles within the cuvette system at 800 nm, and η_T_ is the photothermal conversion efficiency.

$Q_{I}=hA\left( T-T_{amb} \right)-Q_{O}$ (**Eq. S4**)

$Q_{I}=I(1-{10}^{-A_{\lambda}})\eta_{T}$ (**Eq. S5**)

*Targeted HA-BSe NP Ablation of CT26 Colorectal Cancer Cells in Tumor Organoids (1 hr Diffusion)*

Tumor organoids were seeded in a 96-well plate such that the geometry formed is semi-ellipsoidal with its planar surface in direct contact with the plastic well and its curved surfaced exposed as previously described. Organoids were allowed to incubate for 24 hours and then exposed to no nanoparticles, BSe NPs (5.225*10^12^ particles/ mL), or HA-BSe NPs (5.225*10^12^ particles/ mL) suspended in DMEM without phenol red and incubated for 1 hour. Organoids were then washed twice with HEPES-buffered saline (250 mM) + 0.1% BSA to remove nanoparticle aggregates and then immediately treated with an 800 nm continuous wave laser at 5W for 36s with a 1 cm^2^ spot size (K-Cube, Summus Medical Laser) in 100 µL of nanoparticle-free DMEM without phenol red. Twenty-four hours following laser ablation, organoid viability was assessed using CellTiter-Glo 3D Cell Viability Assay (Promega), where luminescence was measured in a Varioskan Lux plate reader (Thermo Fisher).

**Supplemental Results**

Electrostatic layer-by-layer deposition of HA was monitored using DLS and zeta potential measurement for HDAPPs (**Supplementary** **Figure S1**) and BSe NPs (**Supplementary** **Figure S2**). Layer-by-layer Deposition of HA-HDAPPs indicates an iterative increase in the hydrodynamic diameter of HDAPPs with the coating of chitosan, and subsequently, HA, as measured by DLS, resulting in particles with an average 189 nm diameter. The zeta potential shifts from negative for uncoated HDAPPs to positive when coated with chitosan and back to negative when coated with HA, resulting in HA-HDAPPs with a zeta potential of -28.2 mV. HA-BSe NP coating was only observed before and after the full coating process, where increases in size indicate a successful coating, and there was no difference in the zeta potential of HA coated BSe NPs compared to uncoated. Increases in size were also observed when the particles were imaged by TEM and measured by NTA (HDAPPs: 85.1 nm; HA-HDAPPs: 134.2 nm) (**Supplementary** **Figure S3**). NTA size of HA-HDAPPs collected in fluorescence mode (133.9 nm) displayed strong agreement with that of the scattering mode (134.2 nm), indicating that the nanoparticles presenting with fluorescent imaging capacity were in fact the isolated HA-HDAPPs and not two separate nanoparticle populations (**Supplementary** **Figure S3C**).


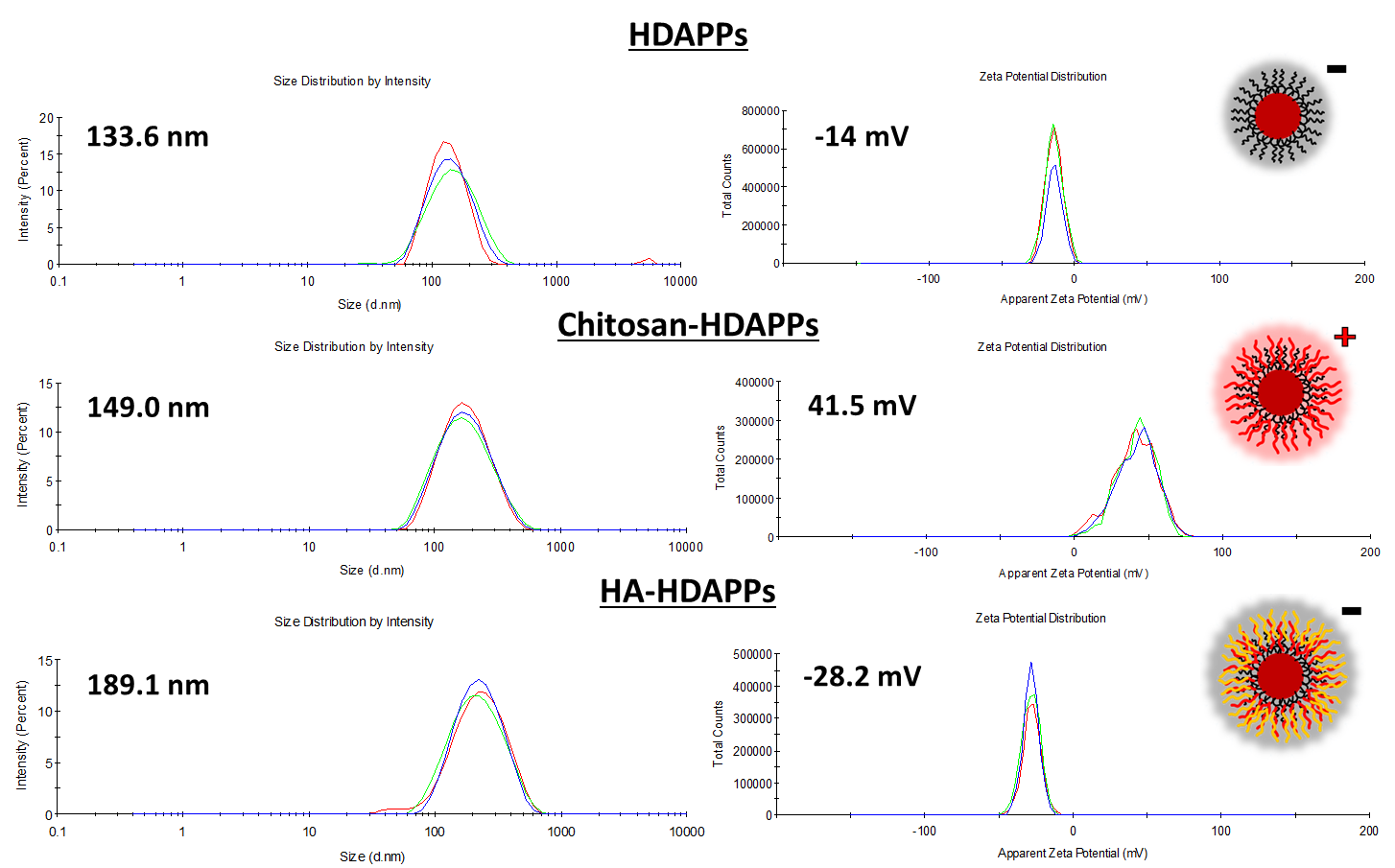


*Supplementary Figure S1:* Dynamic light scattering and zeta potential measurements of HDAPPs during the coating process.


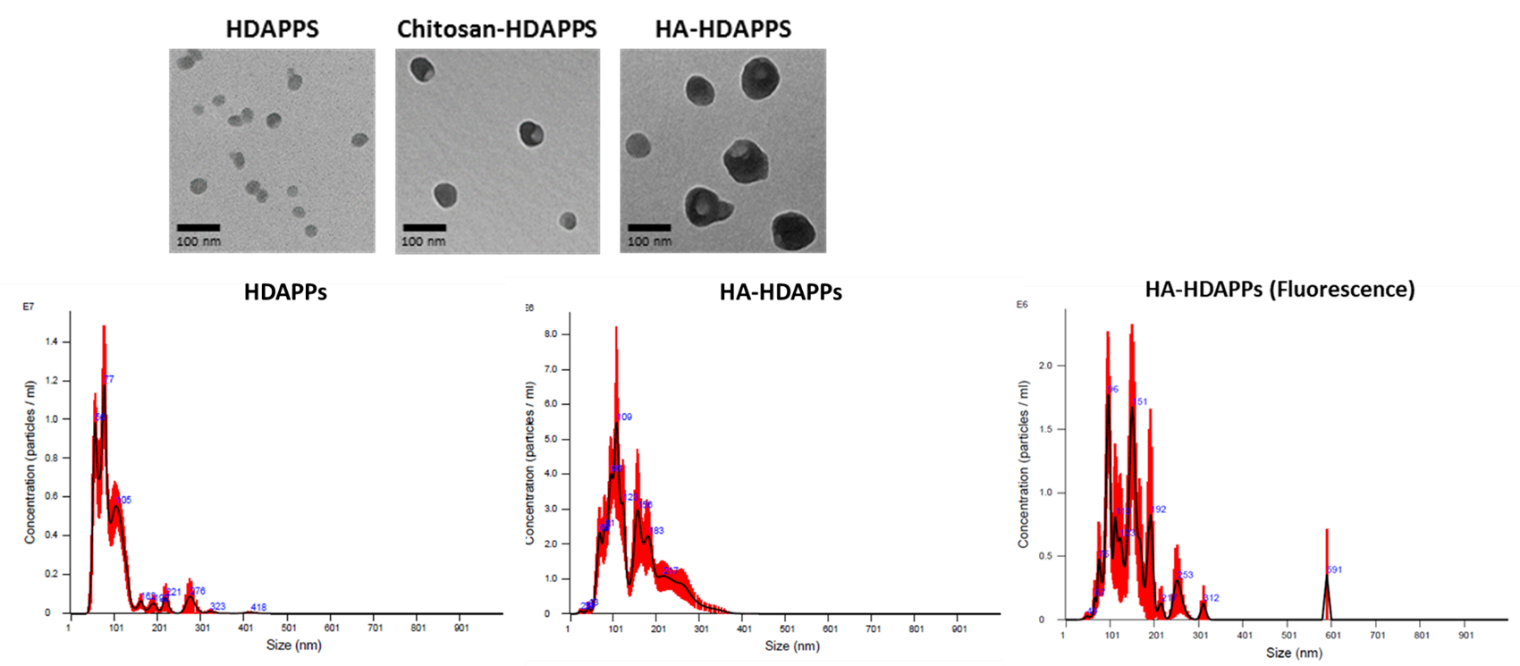


**A**

**B**

**C**

*Supplementary Figure S2:* TEM top images) indicate the spherical nature of the HDAPPs. NTA measurements in scattering mode (A and B) indicate an increase in size with coating. NTA analysis in fluorescence mode confirms the increase in size.


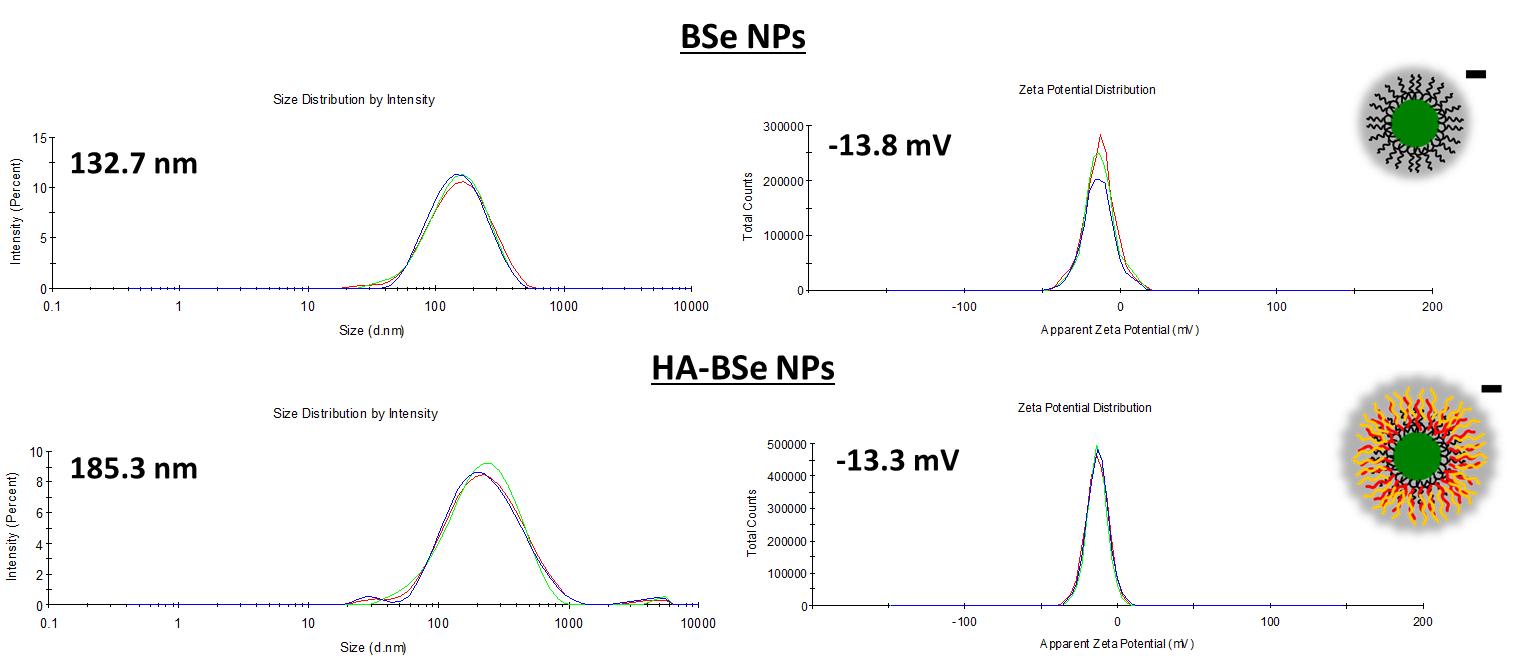


*Supplementary Figure S3:* Dynamic light scattering and zeta potential measurements of BSe NPs after coating with HA.

The temperature rise of increasing concentrations of HDAPPs exposed to 3 W, 60 s of 800nm laser exposure was evaluated, as shown in **Supplementary Figure S4**. The rise is very rapid at the onset of laser exposure and none of the concentrations reach thermal equilibrium. The photothermal conversion efficiency of each nanoparticle was measured according to the protocol outlined by Roper et al. and provided in section 3.2.^2^ Photothermal conversion efficiency (PTCE) values were found to be 51.2%, 57.1%, 53.17%, and 49.8% for HDAPPs, HA-HDAPPs, BSe NPs and HA-BSe NPs, respectively (**Supplementary Figure S5**). This close agreement between all nanoparticle formulations indicates that the BSe polymer PTCE is minimally effected by either coating or core formulation.

**
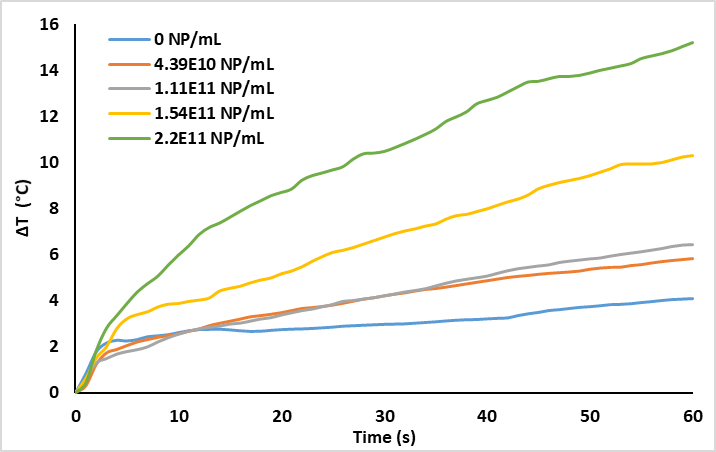
**

*Supplementary Figure S4*: Temperature change over time of a 200 µl volume of HDAPPs at increasing concentrations. Reprint permission provided by Taylor and Francis.


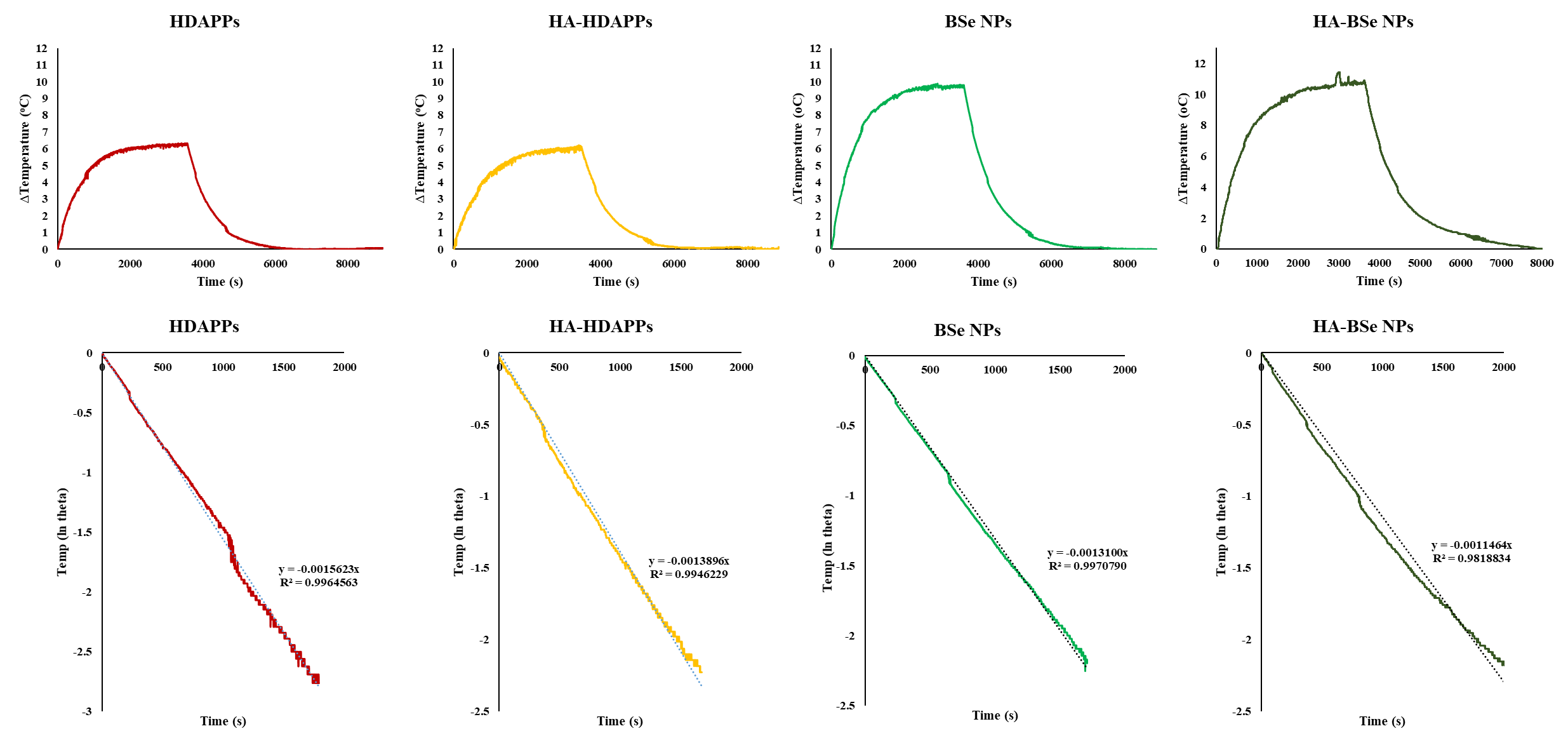


*Supplementary Figure S5*: Photothermal conversion efficiency measurements were calculated using the heating and cooling plots for each nanoparticle (top) with plots of transformed cooling data (bottom) used to calculate τ_s_ (Eq. S3).

To confirm that nanoparticle adherence to the tissue culture plastic was not a factor for the observed increase in HA-HDAPPs binding to cells, images of wells without cells were taken, and show no non-specific binding to the well plastic (**Supplementary Figure S6**). Only for cells treated with HA-HDAPPs was the fluoresnce signaturee of the nanoparticles able to be observed, indicating minimal or no binding of non-funationalized HDAPPs to CT26 cells. To determine the statistical significance between HDAPPs and HA-HDAPPs binding, the linear curves were evalauted in MATLAB, using ANCOVA analysis, bound nanoparticles as the dependent variable and NP/mL as the covariate. As shown in **Supplementary Figure S7**, there is a statistical difference in the slopes (p = 0.0012; α=0.05). ANCOVA analysis also determined that the coating status is the explanatory factor in the difference of the slopes.


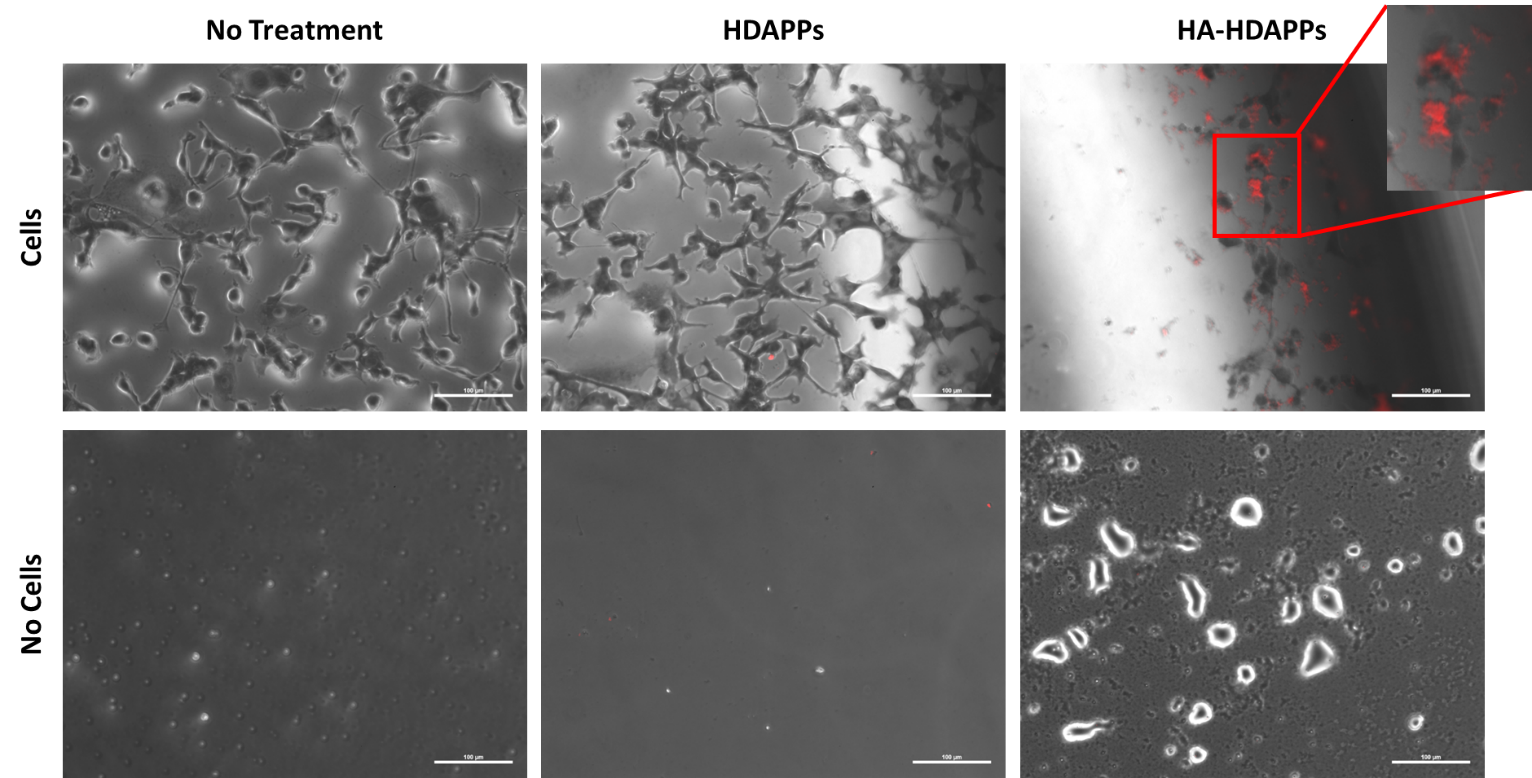


*Supplementary Figure S6*: Fluorescence and bright field microscopy demonstrate HDAPPs binding to cells (upper panels) or adherent to tissue culture plastic (lower panels)


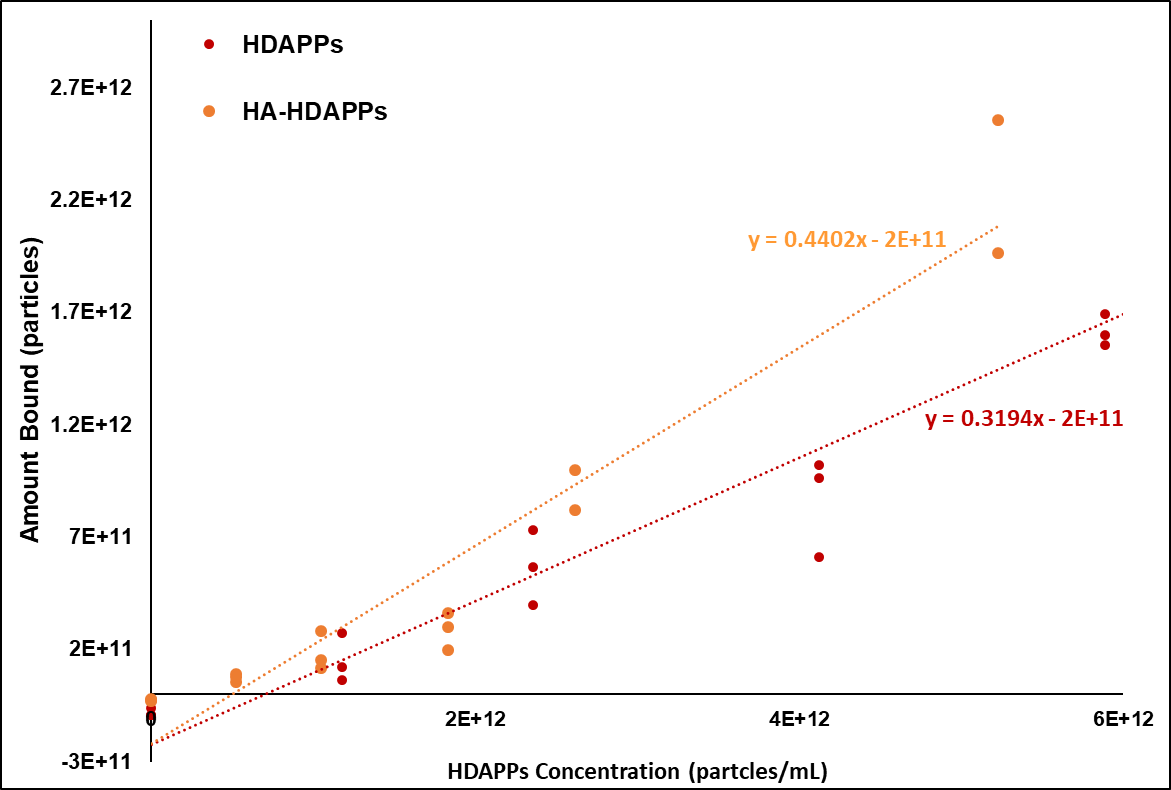


*Supplementary Figure S7*: Linear plot of HDAPPs and HA-HDAPPs binding, used for evaluating statistical variances in bind between the two groups.

To evaluate the correlation between concentration of HDAPPs that diffuse into the organoid and the temperatures that may be attained with laser exposure, 5.225*10^12^ NP/ mL were incubated with non-cell containing organoids and the fluorescence intensity was measured at 0, 4, 12 and 24 hr (**Supplementary Figure S8**). Standard curves developed using organoids developed with specific concentrations of HDAPPs were used to determine the nanoparticle concentration that diffuse into organoids over time. HDAPPs concentration was then used to predict the thermal dose using Figure 1D.

*Supplementary Figure S8*: HDAPPs and HA-HDAPPs diffusion into an organoid was determined by fluorescence and can be used to predict the maximum temperature that CT26 cells would experience based on the concentration of nanoparticles.

An ablation assay of CT26 CRC cells in 2D culture using HDAPPs and HA-HDAPPs was performed according to the protocol outlined in the methods, with a 1hr binding incubation as had been used for HA BSe NPs. No significant decrease in viability was detected within any group following binding and exposure to laser in 2D culture (**Supplementary Figure S9**). This result, plus the binding data and heat curve data from Figures 2A and 1B, support the need to use HA BSe NPs for optimal photothermal therapy.


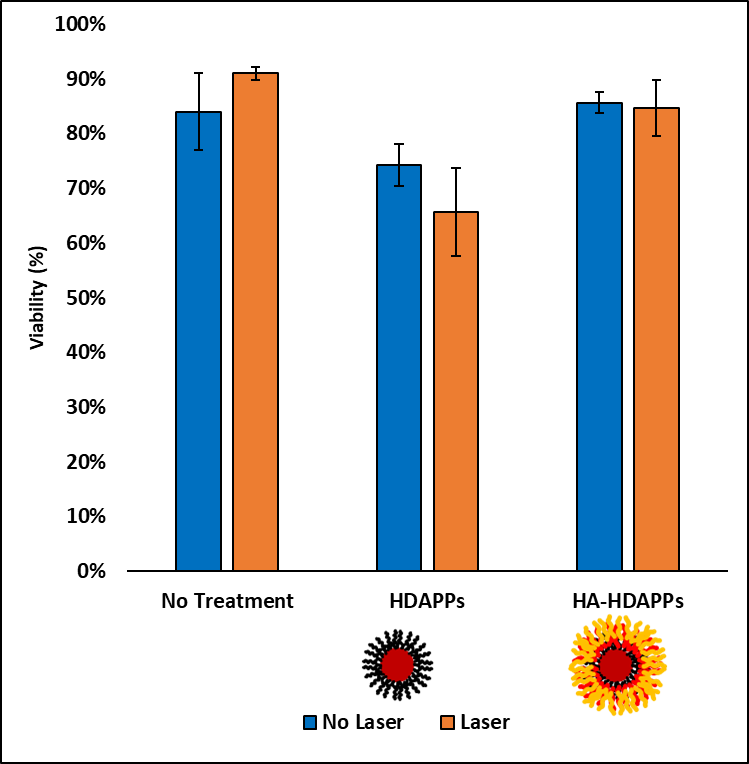


*Supplementary Figure S9:* HDAPPs show no reduction in cell viability in 2D culture, either uncoated or coated with HA.

Following successful ablation of CT26 CRC cells with HA-BSe NPs in 2D culture, a 1hr incubation was used to evaluate the potential for 3D photothermal ablation by diffusion. However, no reduction in viability was observed (**Supplementary Figure S10**). This result was likely due to insufficient diffusion time being allowed to reach ablative thresholds within the organoid matrices.


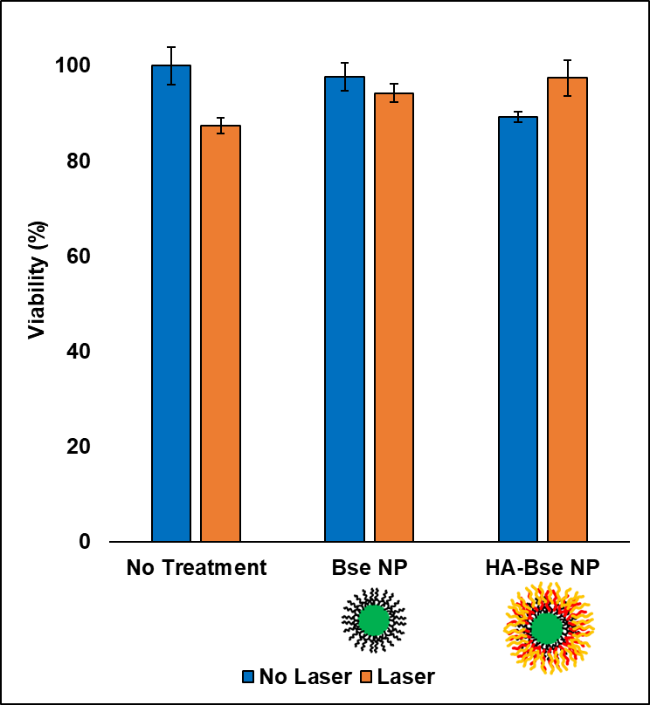


*Supplementary Figure S10:* A 1hr incubation of BSe NPs, similar to the 2D methodology, either with or without HA, does not result in reduced cell viability following organoid exposure to laser stimulation.

**Supplementary Table S1**

| Table 1: Diffusion Model Parameters | | | |
| --- | --- | --- | --- |
| Hyaluronic Acid^3,4^ | r_f_ | 81 | nm |
|  | ν | 0.547 | mL/g |
|  | *φ* | 0.0003 |  |
|  |  |  |  |
| Collagen^5,6^ | r_f_ | 20 | nm |
|  | ν | 1.89 | mL/g |
|  | *φ* | 0.0078 |  |
|  |  |  |  |
| Weighted Average | r_f_ | 22.06 | nm |
|  | *φ_interstitial_* | 0.0081 |  |
|  |  |  |  |
| Stokes Parameters^6^ | k_B_ | 1.38*10^-23^ | m^2^kg/(s^2^K) |
|  | T | 310.15 | K |
|  | η | 0.000818 | Ns/m^2^ |
|  | r_p_ (HDAPPs) | 66.8 | nm |
|  | r_p_ (HA-HDAPPs) | 94.55 | nm |
|  |  |  |  |
| HDAPPs | D_0_ | 4.16*10^-12^ | m^2^/s |
|  | D_int_ | 3.16*10^-12^ | m^2^/s |
|  |  |  |  |
| HA-HDAPPs | D_0_ | 2.94*10^-12^ | m^2^/s |
|  | D_int_ | 2.00*10^-12^ | m^2^/s |
|  |  |  |  |
| Time Parameters | t | 86400 | s |
|  | Δt | 1 | s |
|  |  |  |  |
| Organoid Geometry (Semi-Ellipsoid) | Volume | 6.9 | mm^3^ |
|  | a,c-semiaxes | 2.02 | mm |
|  | b-semiaxis | 0.81 | mm |
|  |  |  |  |
| Concentration Boundary Conditions | Ellipsoid Surface (HDAPPs) | 1.18*10^11^ | NP/mL |
|  | Ellipsoid Surface (HA-HDAPPs) | 1.02*10^11^ | NP/mL |
|  | Well Contact | 0 | NP/mL |

**Supplementary References**

1 Yvon, H. J. A guide to recording fluorescence quantum yields. *HORIBA Jobin Yvon Inc.: Middlesex, UK* (2012).

2 Roper, D. K., Ahn, W. & Hoepfner, M. Microscale heat transfer transduced by surface plasmon resonant gold nanoparticles. *The Journal of Physical Chemistry C* **111**, 3636-3641 (2007).

3 La Gatta, A., De Rosa, M., Marzaioli, I., Busico, T. & Schiraldi, C. A complete hyaluronan hydrodynamic characterization using a size exclusion chromatography–triple detector array system during in vitro enzymatic degradation. *Analytical biochemistry* **404**, 21-29 (2010).

4 Gómez-Alejandre, S., de la Blanca, E. S., de Usera, C. A., Rey-Stolle, M. & Hernández-Fuentes, I. Partial specific volume of hyaluronic acid in different media and conditions. *International journal of biological macromolecules* **27**, 287-290 (2000).

5 Levick, J. Flow through interstitium and other fibrous matrices. *Quarterly Journal of Experimental Physiology: Translation and Integration* **72**, 409-437 (1987).

6 Gao, Y. *et al.* Predictive models of diffusive nanoparticle transport in 3-dimensional tumor cell spheroids. *The AAPS journal* **15**, 816-831 (2013).
